# Variation of skin hydration profile with biophysical factors and lifestyle revealed by *in vivo* terahertz sensing

**DOI:** 10.1364/BOE.527731

**Published:** 2024-08-13

**Authors:** Xuefei Ding, A. I. Hernandez-Serrano, Jacob J. Young, Emma Pickwell-MacPherson

**Affiliations:** 1 Department of Physics, University of Warwick, Gibbet Hill Road, Coventry CV4 7AL, UK; 2 Institute of Applied and Translational Technologies in Surgery, University Hospitals Coventry and Warwickshire, UK

## Abstract

The skin, being the body’s largest organ, plays a pivotal role in protecting the body against dangerous external factors. The maintenance of adequate hydration levels is essential for the skin to fulfill this protective function. However, skin hydration depends upon different biophysical factors and lifestyles, such as ethnicity, sex, age, water consumption, and many more. Consequently, methods to assess skin hydration in a precise and non-invasive manner are in high demand. In this paper, using a portable and handheld terahertz (THz) probe, we systematically examine the correlation between diverse biophysical factors and skin hydration profile in a population exceeding 300 participants. Through comparative analysis of THz light reflected from the skin against a dielectric model, we successfully extracted the thickness and hydration percentage of the outermost layer of the epidermis, the stratum corneum (SC). Our findings indicate that SC hydration and thickness are associated with variables such as daily water consumption, age, drinking coffee, and exercise. Additionally, our measurements reveal distinctions in the skin’s hydration properties concerning susceptibility to UV-induced effects by bringing in the Fitzpatrick skin types. This THz-based technique holds the potential for facile integration into clinical settings for the evaluation and diagnosis of various skin-related conditions.

## 1. Introduction

Skin is the largest organ in the human body and serves as both a protective barrier against external pathogens, and to prevent excessive fluid loss. It is essential for the skin to maintain an appropriate amount of water to function normally and changes in skin hydration can be used as an index for early diagnosis of skin conditions and skin cancers [1,2]. However, the hydration status of the skin can also be altered by various inherent biophysical factors and lifestyle, including ethnicity, Fitzpatrick skin type, sex, age, occupation, exercise and water consumption [3]. Therefore, it is of interest to study the influence of these factors on the skin properties in order to aid the development of early diagnosis techniques for skin diseases [4].

Ethnicity affects an individual’s skin tone by the different levels of chromophores induced [5]. Additionally, the natural hydration and thickness of the stratum corneum (SC) vary among ethnic groups: in general the White and the Black ethnic groups possess drier skin than the Chinese due to lower natural moisturizing factors in the SC [6,7]. The Fitzpatrick scale divides people into six different skin types based on their skin’s reaction to the sun. This can be used as an effective indicator for differences in skin tone, since people from the same ethnic group may have variations in skin type [8,9]. The correlation between sex and skin hydration and SC thickness is not as clear, where the impact on skin induced by this factor is often outweighed by other individual variations [4,10,11]. Some studies reported that the skin hydration level has a negative correlation with age as a result of the decreasing natural moisturizing factors in skin [4,10], while others presented opposite results [12], due to diverse ways of grouping the participants into different age groups. For example, Man *et al.* showed that the forearm skin hydration for males between the age of 0-50 years old increased with age, with the highest skin hydration observed in the age group of 36-50 years old, and then the hydration decreased significantly for males over 70 years old [10]. While in [12], their subjects were divided into two groups with the younger group in the age of 20-24 years old and the elder group in the age of 60-68 old years, and they found slightly higher epidermal hydration in the elder group compared to the younger group. The volume of daily fluid consumption is a main parameter to look at when studying the relationship between lifestyle and skin condition. Boelsma *et al.* reported a weak positive correlation between daily fluid intake and the SC hydration level [13]. Some studies showed that an additional water intake on top of individuals’ regular diet on a daily basis for over 30 days would result in an increase in the SC hydration [14,15].

In existing studies investigating factors affecting the biophysical properties of skin, skin hydration is mostly measured through the skin capacitance with a corneometer, which is compact, portable and inexpensive. However, limitations of the corneometer include that it is easily influenced by environmental factors and it only provides a relative value for the skin hydration given in a scaled measurement of the capacitance [16,17]. Terahertz (THz) light has a frequency range between 0.1-10 THz (1 THz=10^12^ Hz), which is non-ionizing with low photon energies and is strongly absorbed by water. This means THz light has a high sensitivity to changes in the water content of a sample. Such advantageous features make THz spectroscopy a potential candidate for biomedical examinations, including *ex vivo* measurements to distinguish excised cancerous tissues [18–22] and *in vivo* measurements for real-time, non-invasive monitoring of skin hydration [23–28]. Pilot *in vivo* THz studies have so far mostly been laboratory-based with conventional THz-TDS systems that are bulky in size, and the sample sizes used in the studies are often small. In this study, we perform detailed analysis of *in vivo* data acquired using a portable handheld THz scanner of over 300 participants. This study was conducted in an out-of-the-lab environment and is the largest of its kind hitherto. Full details of the experimental protocol and the probe development are given in Ref. [29]. In this paper we extend the analysis to investigate the impact of different biophysical factors and lifestyle on the initial hydration condition of skin within a diverse population of subjects.

## 2. Methods

### 2.1 Experimental setup and protocols

Ethical approval was acquired from the Biomedical Scientific Ethics Committee, BSREC, (REGO-2018-2273 AM03) prior to the study. The measurements were taken in an out-of-the-lab environment over four days with the ambient temperature at 16 ^∘^*C*, 19 ^∘^*C*, 17 ^∘^*C*, 5 ^∘^*C*, and the ambient humidity at 70%, 61%, 62%, 78% respectively. Participants were asked to roll up their sleeves upon arrival to let their volar forearm acclimatize to the ambient room conditions. Then they were provided with an information sheet (including details of the protocol of the study) and their consent of participation was acquired. Participants were also asked to fill out the questionnaire, which included questions regarding their biophysical factors, lifestyle and details of any relevant skin conditions or medical conditions. Table 1 presents a list of the main categories involved in the questionnaire regarding the participants’ biophysical factors and lifestyle along with the number of participants in each category. 19 participants who failed to input their correct measurement IDs in the questionnaires have been excluded from this study, since their THz measurements can not be associated with their personal information without the correct measurement IDs.

**Table 1.** Main categories in the questionnaire and the number of participants in each category.

Category		No. of Participants
**Sex**	Female	103
	Male	192
	Prefer not to tell	3

**Year of Birth**	2000 – 2004	118
	1990 – 1999	123
	1980 – 1989	26
	1970 – 1979	17
	1956 – 1967	14

**Dominant Hand**	Right-handed	266
	Left-handed	32

**Ethnicity**	White	214
	Chinese Black	20 11
	Others	53

**Fitzpatrick Skin Type**	Type I	19
	Type II	59
	Type III	132
	Type IV	62
	Type V	18
	Type VI	8

**Skin Condition**	Healthy skin	242
	Dry skin	53
	Other skin conditions	3

**Water Consumption**	Less than 1L	40
	1L to 2L	121
	2L to 3L	114
	More than 3L	23

**Drinking Coffee**	No	129
	More than 2 hours ago	72
	Within the last 2 hours	46
	Within the last 1 hour	24
	Within the last 30 minutes	27

**Exercising**	No	212
	More than 2 hours ago	55
	Within the last 2 hours	21
	Within the last 1 hour	3
	Within the last 30 minutes	7

**Fig. 1.**
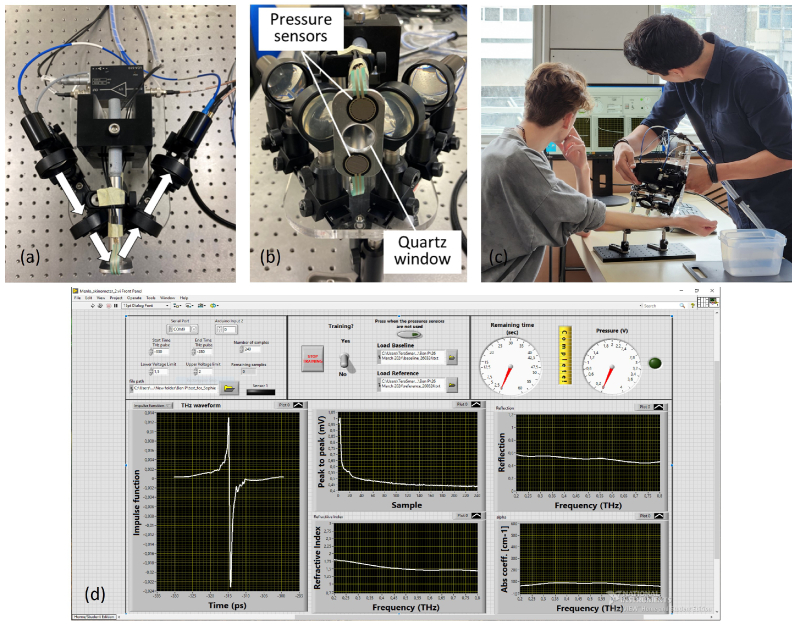
(a-b) The THz handheld scanner. (c) A researcher operating the handheld scanner for *in vivo* THz measurement of the volar forearm of a participant during the study. (d) A screenshot of the self-developed software after one measurement.

A continuous THz measurement was then taken on the volar forearm with a portable handheld THz scanner for 60 seconds, at a recording rate of 4 pulses per second. As shown in Fig. 1(a-b), the handheld scanner is built with a fibre coupled THz emitter and a detector from the TeraSmart THz-TDS spectrometer from Menlo Systems and arranged in a reflection setup with a 30^∘^ incident angle. The THz beam spot is elliptical with axes of 5 mm and 10 mm when measured in reflection setup. A quartz sampling window with a diameter of 2 cm serves to both align the THz light to the skin surface, and also to flatten the skin during a measurement. Two pressure sensors are attached next to the quartz window to ensure a steady and consistent force is applied (0.6-1.1 *N/cm*^2^) to the skin during measurements [30,31]. The same handheld scanner was used as reported in [29]. The handheld scanner has been demonstrated to measure the dielectric properties of skin in excellent agreement with other commercial THz systems, while also having enhanced stability due to its superior mechanical rigidity [29]. This allows consistent alignment of the quartz window. The start of the measurement was triggered by the pressure sensors when the skin was in good contact with the quartz window, after which 240 THz scans were recorded during the 60 second measurement, which would allow us to see clear occlusion effect on skin [32]. In addition, a single measurement of air was taken as the reference signal and a single measurement of an identical quartz on top of the quartz window was taken as the baseline signal, both of which assist in the data processing and calibration of the raw THz signals [27]. Figure 1(c) shows a researcher using the handheld THz scanner to measure a participant’s volar forearm: the self-developed software displays the real-time reflected THz signal, the contact pressure and the occlusion curve on the monitor (Fig. 1(d)).

### 2.2 Skin modelling approach

**Fig. 2.**
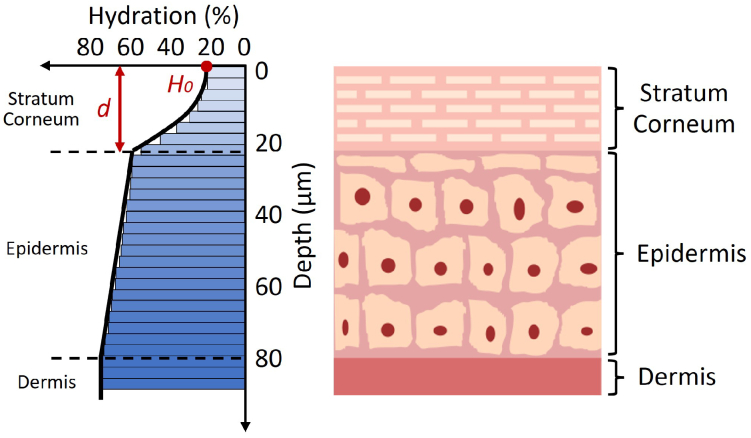
A diagram showing the skin layers (stratum corneum (SC), epidermis and dermis) and the skin hydration change as a function of depth. The skin hydration change follows a parabolic trend in the SC, a linear increase in the epidermis until reaching about 80%. The red dot indicates the SC surface Hydration $\left(H_{0}\right)$ , and the red arrow indicates the SC thickness (*d*).

Skin can be separated into three main layers: the epidermis, dermis and subcutaneous tissue. The stratum corneum (SC) is the outermost layer of the epidermis and contains a gradient of water concentration which is different from the rest of the epidermis. Therefore, here we model the SC and the remaining epidermis separately and refer to the remaining epidermis as "epidermis". Skin has a high water content, normally ranging from 20% to 80% and since water strongly attenuates THz light, we are to date only able to probe the SC and part of the epidermis layer. Figure 2 illustrates our model of the skin structure and the corresponding water concentration. Skin also consists of collagen, elastin and other proteins [33]. Many THz *in vivo* studies looking at the skin hydration change have applied a single-layer or a two-layer model to simulate the skin structure [27,30,32]. The single-layer model simplifies the skin by assuming it is comprised of 1 semi-infinite homogeneous layer. This layer is assumed to have uniform water distribution. Whereas, the two-layer model separates the skin into the SC layer and the epidermis layer with each layer having a different water concentration, this allows for some depth dependant water content in the skin, but still heavily simplifies the system. Confocal Raman spectroscopy studies have shown that the water concentration in skin is depth dependent according to a parabolic function in the SC, a slightly increasing linear function in the epidermis until reaching about 80% water, and then holds a constant value in the dermis [34,35].

Therefore, in this work we use a stratified media model to simulate the reflected THz beam from skin with a more realistic hydration profile similar to the one observed in Raman spectroscopy [36]. This composite model describes a plane wave of THz light reflected off an effective medium that has stratified dielectric permittivity. This model separates the skin into multiple thin layers of 2 *μ*m to approximate the continuous water concentration change. The composition of skin is seen as a binary mixture of water content and dry biological background, therefore in each stratified layer the effective permittivity is associated with the water concentration through the Bruggeman effective medium theory (EMT) model, and is written as:

(1) $$\eta_{1}\frac{\varepsilon_{1}-\varepsilon_{eff}}{\varepsilon_{1}+2\varepsilon_{eff}}+\eta_{2}\frac{\varepsilon_{2}-\varepsilon_{eff}}{\varepsilon_{2}+2\varepsilon_{eff}}=0$$

where $\eta_{1}$ and $\varepsilon_{1}$ are the volume fraction and permittivity of the water content, $\eta_{2}$ and $\varepsilon_{2}$ are the volume fraction and permittivity of the dry biological background, and $\eta_{1}+\eta_{2}=1$ . $\varepsilon_{eff}$ is the effective permittivity of the whole composite system. The permittivity of water is described by a double Debye model with the parameters reported in the literature $\left(\varepsilon_{s}=78.4,\varepsilon_{2}=4.9,\varepsilon_{\infty }=3.5,\tau_{1}=8.2\times 10^{-12}\;s,\tau_{2}=0.18\times 10^{-12}\;s\right)$ [37], and the permittivity of the dry biological background is approximated by the measured result of dehydrated porcine skin (n=1.20) [36]. The effective permittivity of the skin system is therefore directly associated with the volume fraction of the water content.

The propagation of the wave in each layer is characterized by a longitudinal propagation constant $\left(k_{m}\right)$ and a characteristic impedance $\left(\zeta_{m}\right)$ given by Eqs. (2) and (3). Consequently, the effective impedance looking down at the top of the *m*-th layer can be calculated in a recursive fashion through Eq. (4).

(2) $$k_{m}=\omega \sqrt{\varepsilon_{0}\mu_{0}}\sqrt{\varepsilon_{m}-sin^{2}\theta }$$

(3) $$\zeta_{m}=\frac{\omega \mu_{m}}{k_{m}}$$

(4) $$Z_{m}=\zeta_{m}\frac{Z_{m+1}+i\zeta_{m}tan(k_{m}t_{m})}{\zeta_{m}+iZ_{m+1}tan(k_{m}t_{m})}$$

Here $\varepsilon_{m}$ and $\mu_{m}$ represent the effective permittivity and permeability of each layer, and $t_{m}$ is the layer thickness. θ is the incident angle of the wave on the surface of the system. Through iteration of the former equations, reflections from this multilayer system can be summed up from the deepest layer all the way to the surface, resulting in the effective reflection coefficient seen from the top of the system given by Eq. (5).

(5) $$\Gamma_{0}=\frac{Z_{1}-\zeta_{0}}{Z_{1}+\zeta_{0}}$$

**Fig. 3.**
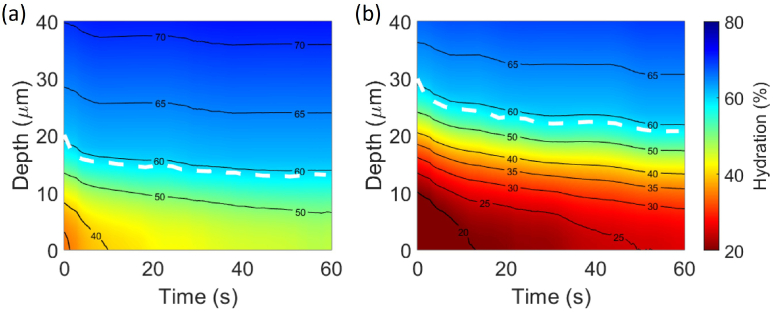
An example of skin hydration as a function of depth into the skin and occlusion time for two participants, with (a) more hydrated skin and (b) drier skin. The solid black lines are the contour lines of the hydration and the dashed white lines indicate the boundary between SC and epidermis. The colour bar for both figures is given on the right.

Results from the confocal Raman spectroscopy reveal that when the skin is hydrated, the main changes appear in the SC surface hydration $\left(H_{0}\right)$ and SC thickness (d) [34]. Therefore, the water gradient inside the skin can be represented by a depth dependent function with $H_{0}$ and *d* as unknown variables, which can then be used to calculate the reflectivity of the skin using the stratified media model and the Bruggeman EMT model. Other variables in the skin model (as shown in Fig. 2) are kept as constants, including hydration level at the SC-epidermis surface, hydration level at the epidermis-dermis surface, epidermis thickness and dermis thickness. The measured THz reflectivity from *in vivo* skin measurement can be acquired according to the bulk-sample treatment for THz reflection geometry as described in section 2.4. By fitting the calculated reflectivity to the measured reflectivity, the SC surface hydration and SC thickness can be extracted. Figure 3 shows an example of the skin hydration of two individuals changing as a function of depth into the skin (y-axis) and occlusion time (x-axis). This colourmap is fully determined by the extracted SC surface hydration $\left(H_{0}\right)$ and SC thickness (d) as a function of the occlusion time, therefore in the Results section we only focus on $H_{0}$ and *d* and compare them among a large cohort of individuals. Figure 3(a) is for a participant with more hydrated skin as the colourmap has shades corresponding to hydration of 50% and above throughout the measurement for depths beyond about 15 *μ*m. In contrast Fig. 3(b) is from a participant with drier skin and the colourmap shows regions of SC with hydration lower than 30% (red tones) throughout the measurement, and the SC only reaches hydration above 50% at depths beyond about 20 *μ*m.

### 2.3 Simulation and model fitting

To understand the influence of skin properties on the THz reflection coefficient, simulations have been conducted on the reflectivity change subject to different SC surface hydration and SC thicknesses. The incident angle in the simulations is set to be the same as the experimental setup, which is 30^∘^. The hydration level is set to be 75% at the SC-epidermis interface and 90% at the epidermis-dermis interface. The epidermis and dermis thickness are set to be 80 *μ*m and 100 *μ*m [34] respectively, though in practice the THz light will not penetrate deeply enough to reach the dermis. The SC thickness is kept at 20 *μ*m when looking at the impact of SC surface hydration, and the SC surface hydration level is kept at 30% during the variation of the SC thickness. Simulations are conducted in the frequency range of 0.3-0.8 THz, which is the same frequency range for processing the experimental results of skin measured with the THz handheld scanner to avoid large scattering effect in the higher frequencies and low signal-to-noise ratio in the lower frequencies.

**Fig. 4.**
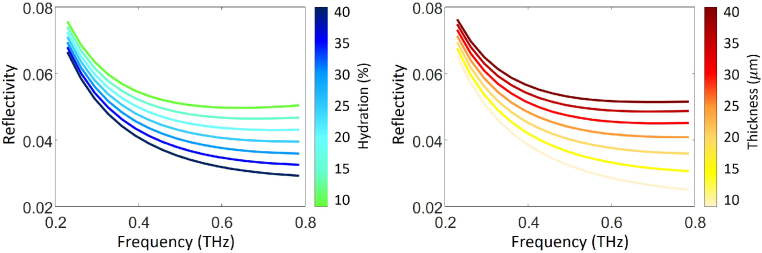
Simulation of the reflectivity changing with (a) the SC hydration and (b) the SC thickness. The epidermis surface hydration is 75%, the epidermis thickness is 80 *μ*m, the dermis hydration is at 90% and the dermis thickness is 100 *μ*m. (a) The SC thickness is kept at 20 *μ*m while the SC surface hydration changes in 10-40%. (b) The SC surface hydration is kept at 30% while the SC thickness changes in 10-40 *μ*m.

According to Fig. 4, the reflectivity curve shifts down when the SC surface hydration increases from 10% to 40%, whereas it rises upwards along with the increase of the SC thickness from 10 *μ*m to 40 *μ*m. The strength of influence of the two variables seems to be comparable given the similar amount of variation in the reflectivity curve induced by a unit change in each variable.

**Fig. 5.**
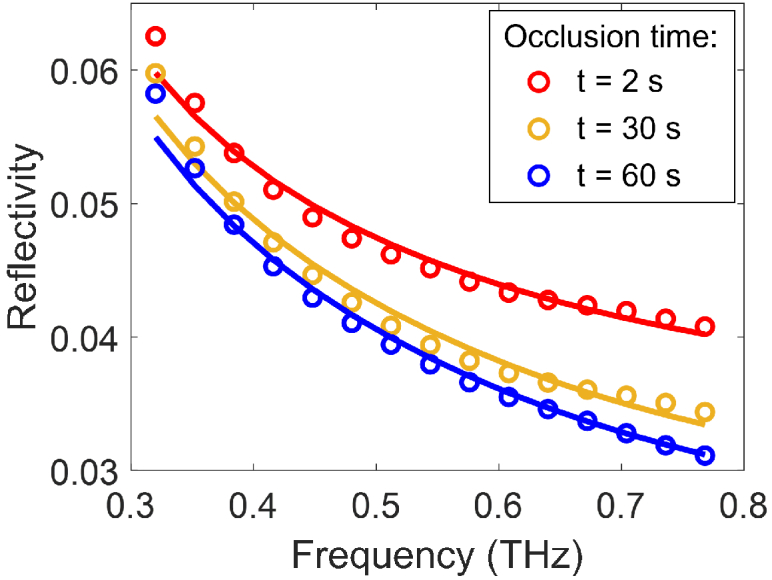
An example of fitting the calculated reflectivity to the measured reflectivity of one individual. Dots represent the measured reflectivity and solid lines represent the fitted reflectivity. Different colours indicate the reflectivity of skin measured at different time into occlusion, with red, yellow and blue standing for 2 seconds, 30 seconds and 60 seconds respectively.

Figure 5 presents an example of fitting the calculated reflectivity (solid lines) to the measured reflectivity (circles) of a 60 second measurement of one individual in the range of 0.3 to 0.8 THz. The figure shows the accuracy of fitting to the measured reflectivity with the skin modelling approach described above, and also the variation of the reflectivity curve due to occlusion. As expected, when the skin hydration increases along with the occlusion time, the measured reflectivity curve moves down vertically and the aforementioned skin modelling approach is capable of fitting each reflectivity curve precisely.

## 3. Results

### 3.1 Impact of sex, age and dominant hand on skin properties

Our previous study of the occlusion effect [32] showed that the THz response of the skin depends on how long it has been in contact with the quartz window. Therefore, if just a single time domain pulse were used for analysis, it would be difficult to know at which point in the occlusion process it was measured at. The greatest change in the THz response due to occlusion is in the first 10 seconds and the curve is much flatter after about 40 seconds, therefore by measuring for 60 seconds and looking at the occlusion curve, we are able to make more meaningful comparisons. Details of our robust methods for making relative change comparisons are given in [31] and the calculation of the SC thickness and hydration are given in [29].

As introduced in the previous section, skin surface hydration and SC thickness can be influenced by lifestyle and various biophysical factors. This section studies the influence of sex, age and dominant hand.

**Fig. 6.**
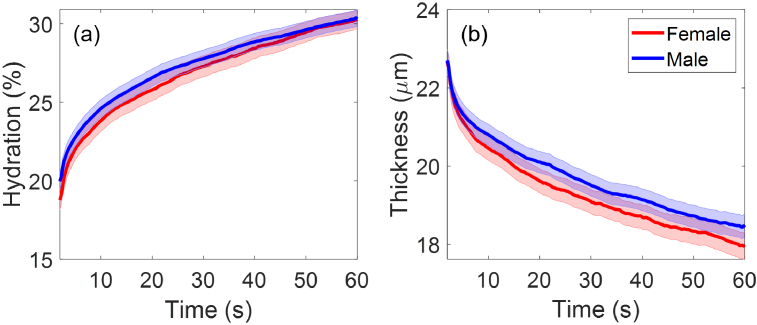
The extracted (a) SC hydration and (b) SC thicknesses as a function of the measurement time compared between female participants (103 people) and male participants (192 people). The solid lines indicate the mean values and the coloured patches show the standard errors of the mean among all the individual results in each category.

Figure 6 presents the extracted SC hydration and thicknesses as a function of the measurement time (2-60 seconds) for all the female participants (103 people) and all the male participants (192 people). From Fig. 6 it can be observed that although the male participants group shows a slightly higher mean value in the SC hydration and SC thickness compared to the female participants group, the error bars of the two groups largely overlap with each other during all the measurement time. Student’s t-tests were performed and results showed that p>0.05 for SC hydration and SC thickness throughout the entire measurement time, therefore it can not be confirmed that there is any significant difference in the skin properties induced by the different sexes. Similar results have also been reported in [3], which stated that the correlation between skin hydration and sex is usually outweighed by individual variation.

**Fig. 7.**
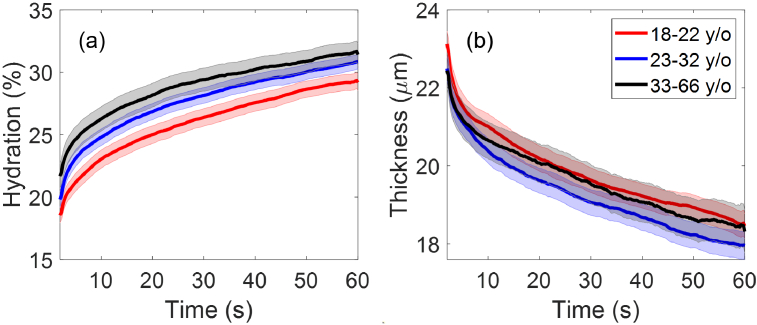
The extracted (a) SC hydration and (b) SC thicknesses as a function of the measurement time compared between participants aged 18-22 years old (118 people), 23-32 years old (123 people) and 33-66 years old (57 people). The solid lines indicate the mean values and the coloured patches show the standard errors of the mean among all the individual results in each category.

**Fig. 8.**
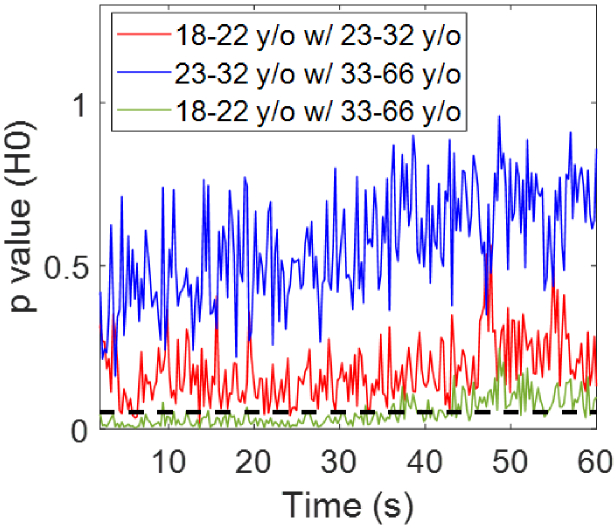
Results of performing one-way ANOVA tests and Tukey’s Honest Significant Difference tests on the SC hydration results of the different age groups. Here y-axis is the p value and x-axis is the measurement time. The solid lines represent the pairwise comparisons between each two groups as shown in the label. The black dashed line indicates where p=0.05 .

Figure 7 shows the extracted SC hydration and thicknesses as a function of the measurement time for participants who were 18-22 years old (born in 2000-2004), 23-32 years old (born in 1990-1999) and the rest of the participants who were 33-66 years old (born in 1956-1989). There are 118, 123 and 57 participants in the aforementioned age groups respectively. It is observed from Fig. 7(a) that in general older participants show higher SC hydration during the entire measurement, where the group of participants aged 33-66 years old have higher mean SC hydration than the group aged 23-32 years old at the time of measurement and the group aged 18-22 years old have the lowest mean SC hydration. In Fig. 7(a), the error bars of the standard error of the mean (SEM) show clear separation between the 18-22 years old group and the other two groups, with slight overlapping between the 23-33 years old group and the 33-66 years old group. In Fig. 7(b), there are some differences in the mean SC thickness between the three age groups, but the SEMs are all overlapping with each other. One-way analysis of variance (ANOVA) tests followed by Tukey’s Honest Significant Difference tests were performed, where p>0.05 for all the pairwise comparisons of SC thickness and the results for SC hydration are presented in Fig. 8.

Figure 8 shows that there are significant pairwise differences (p<0.05) between the age groups of 18-22 years old and 33-66 years old during most of the measurement time up to 40 seconds; significant pairwise differences (p<0.05) only occur between the 18-22 years old group and the 23-32 years old group at 8 time points around 5, 14 and 22 seconds; and there are no significant pairwise differences (p>0.05) between the 23-32 years old group and the 33-66 years old group. Therefore it can be concluded that participants aged 33-66 years old show significantly higher SC hydration than participants aged 18-22 years old during the first 40 seconds of measurement. The average hydration of the older group is higher than the younger group for the whole duration of the measurement (60 seconds) but due to the high standard deviation towards the end of the measurement we have only been able to measure it with statistical significance for the first 40 seconds. The larger standard deviation could be due to participants getting restless and moving more towards the end of the 60 seconds. This is consistent with the literature. For example, Man et al. reported in [10] that within a group of 328 male participants in the age of 0.5-94 years old, the SC hydration on their forearm measured by a corneometer showed that participants within the age groups of 36-50 and 51-70 years old have higher SC hydration compared to those in 13-35 and 0-12 years old.

**Fig. 9.**
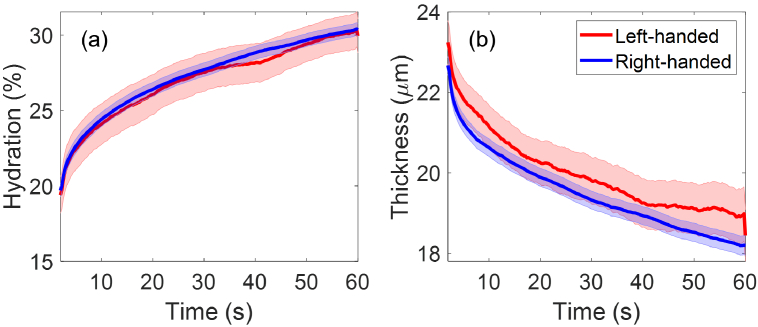
The extracted (a) SC hydration and (b) SC thicknesses as a function of the measurement time compared between the left-handed group (32 people) and the right-handed group (266 people). The solid lines indicate the mean values and the coloured shading shows the standard errors of the mean among all the individual results in each category.

Dominant hand analysis of the extracted SC hydration and thicknesses is presented in Fig. 9, with the left-handed group (32 participants) shown in red solid lines, and the right-handed group (266 participants) in blue. It can be observed from Fig. 9(a) that the two groups have similar mean SC hydration with the SEM error bars fully overlapping with each other. In Fig. 9(b), the left-handed group show a larger mean SC thickness compared to the right-handed group during the entire measurement, but the SEM error bars are strongly overlapping between the two groups. Furthermore, Student’s t-tests showed that p>0.05 for SC hydration and SC thickness between the two groups during the entire measurement time, indicating no significant impact on the skin properties is induced by different dominant hands.

### 3.2 Impact of ethnicity and skin tone on skin properties

**Fig. 10.**
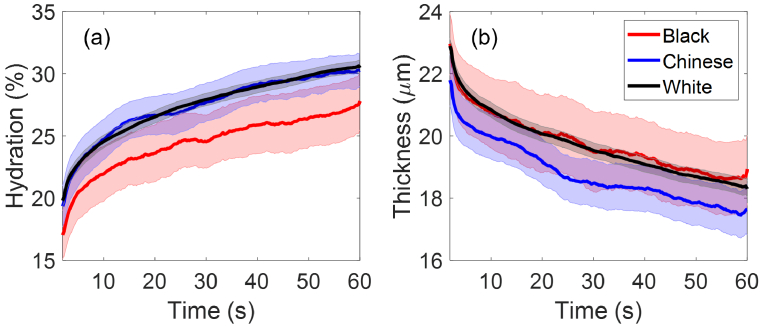
The extracted (a) SC hydration and (b) SC thicknesses as a function of the measurement time compared between the Black ethnic group (11 people), the Chinese ethnic group (20 people) and the White ethnic group (214 people). The solid lines indicate the mean values and the coloured patches show the standard errors of the mean among all the individual results in each category.

This section studies the correlation between individuals’ initial skin properties and their ethnicity and skin tone. Here three main ethnic groups within our recruited participants are investigated: the Black ethnic group (11 participants), the Chinese ethnic group (20 participants) and the White ethnic group (214 participants). From Fig. 10(a) it can be seen that the White ethnic group have comparable mean SC hydration with the Chinese ethnic group with fully overlapping SEM error bars, while the Black ethnic group show the lowest mean SC hydration with the error bars slightly overlapping with the other two groups. In Fig. 10(b), the White ethnic group and the Black ethnic group show identical mean SC thickness with fully overlapping error bars, while the Chinese ethnic group appear to have smaller mean SC thickness with the error bars strongly overlapping with the Black ethnic group and slightly overlapping with the White ethnic group. Results from one-way ANOVA tests and Tukey’s Honest Significant Difference tests showed that p>0.05 for all the pairwise comparisons, which means that although we have identified some trends, there are no statistically significant differences in skin properties between the three ethnic groups. Further study with a larger sample size would be needed to potentially observe statistically significant differences.

**Fig. 11.**
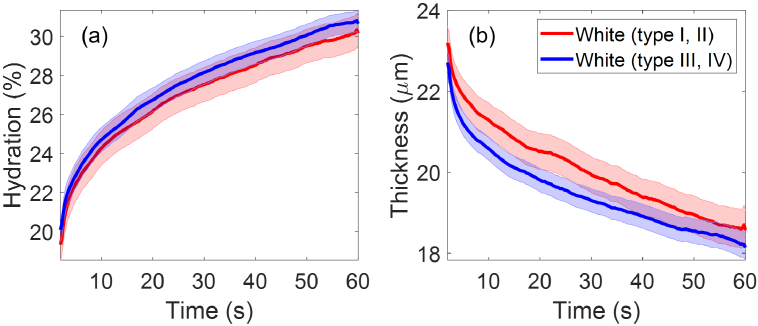
The extracted (a) SC hydration and (b) SC thicknesses as a function of the measurement time compared between participants in the White ethnic group who have type I or type II skin (78 people) and those who have type III or type IV skin (136 people). The solid lines indicate the mean values and the coloured patches show the standard errors of the mean among all the individual results in each category.

Fitzpatrick skin type is a recognized tool for numerically categorizing human skin tone and skin’s response to the sun [8]. Under this scheme, participants in the White ethnic group with different skin tones were divided into several skin types including type I, II, III, IV. For further investigation, 78 participants in the White ethnic group with type I and II skin are compared to the other 136 participants with type III and IV skin. It is then observed in Fig. 11 that the type I-II group have slightly higher mean SC hydration and smaller mean SC thickness compared to the type III-IV group, with strong overlapping in the SEM error bars for the SC hydration and slight overlapping for the SC thickness. Results from Student’s t-tests suggested that p>0.05 for SC hydration during the entire measurement and SC thickness for most of the measurement time except for 3 time points around 6 and 25 seconds into the measurement where p<0.05 . Therefore, we can not draw a conclusion on whether different Fitzpatrick skin types (or different skin tones) have an impact on the initial skin properties. Indeed, within the same ethnic group, people might have different Fitzpatrick skin types, and this makes it difficult to categorize the data into suitable groupings for comparison.

**Fig. 12.**
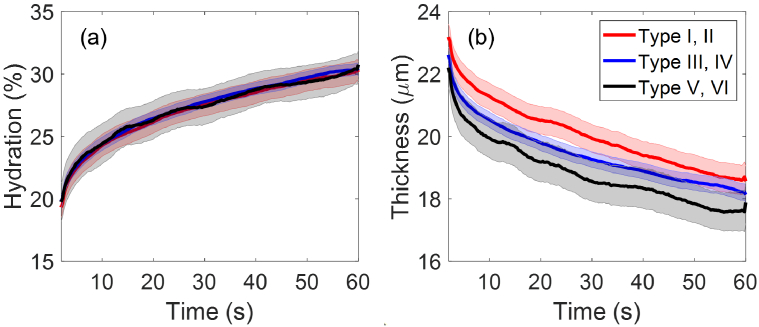
The extracted (a) SC hydration and (b) SC thicknesses as a function of the measurement time compared between participants who have type I or type II skin (78 people), type III or type IV skin (194 people) and those who have type V or type VI skin (26 people). The solid lines indicate the mean values and the coloured patches show the standard errors of the mean among all the individual results in each category.

Within the entire cohort, there are 78 participants with type I-II skin, 194 participants with type III-IV skin and 26 participants with type V-VI skin. As shown in Fig. 12, the three groups with different skin types have overall identical mean SC hydration with fully overlapping SEM error bars, but their SC thicknesses are different with the type I-II group having the largest mean SC thickness and the type V-VI group having the smallest. One-way ANOVA tests and the Tukey’s Honest Significant Difference tests were then performed on the SC thickness and significant pairwise differences (p<0.05) were only found at 25 seconds into measurement for type I-II and type III-IV groups, and at 18 and 25 seconds into measurement for type I-II and type V-VI groups. Therefore, the statistical significance of the impact of Fitzpatrick skin type (or skin tone) on individual’s skin properties is still unclear.

Overall, it is interesting to note that there are no significant differences in hydration or thickness if grouping participants into ethnicity. However, if we look at data across all the Fitzpatrick scales we see that there is a more noticeable difference in skin thickness than hydration, and the difference between type I, II and type V, VI is the most significant (Fig. 12(b)).

### 3.3 Impact of lifestyle on skin properties

Another aspect of factors we are interested in is participants’ lifestyle, including their daily water consumption, coffee consumption and exercise routine, and how these factors affect the participants’ initial skin hydration and thickness.

**Fig. 13.**
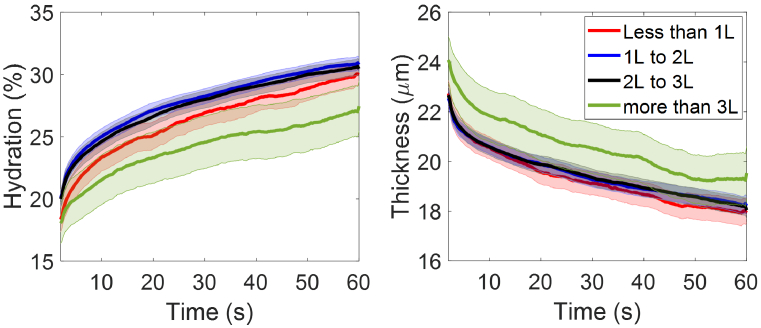
The extracted (a) SC hydration and (b) SC thicknesses as a function of the measurement time compared between participants who drink less than 1 litre of water per day (40 people), 1-2 litres of water per day (121 people), 2-3 litres of water per day (114 people) and those who drink more than 3 litres water per day (23 people). The solid lines indicate the mean values and the coloured patches show the standard errors of the mean among all the individual results in each category.

In our questionnaire, the question regarding participants’ daily water consumption asks the typical amount of water the participant consumes in one day with options of several volume ranges. Within the entire cohort, there are 40 participants who drink less than 1 litre of water per day, 121 participants who drink 1 to 2 litres, 114 participants who drink 2 to 3 litres, and 23 participants who drink more than 3 litres of water per day. As shown in Fig. 13(a), the groups that drink 1 to 2 litres and 2 to 3 litres of water per day show identical and also the highest mean SC hydration compared to the other two groups, and the group that drink more than 3 litres have the lowest mean SC hydration. In Fig. 13(b), the group that drink more than 3 litres of water per day have the largest mean SC thickness, while the other three groups show similar mean SC thickness. However, according to the results from One-way ANOVA tests and the Tukey’s Honest Significant Difference tests, there are no significant pairwise differences (p>0.05) between any two groups for both the SC hydration and SC thickness.

**Fig. 14.**
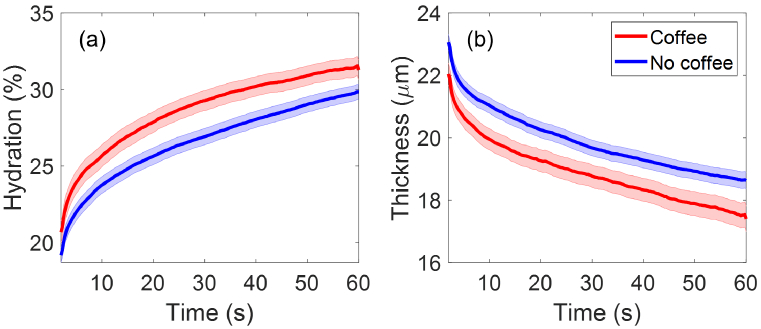
The extracted (a) SC hydration and (b) SC thicknesses as a function of the measurement time compared between participants who have drunk coffee within 2 hours prior to the experiment (97 people) and those who haven’t drunk coffee or have drunk coffee more than 2 hours prior to the experiment (201 people). The solid lines indicate the mean values and the coloured patches show the standard errors of the mean among all the individual results in each category.

**Fig. 15.**
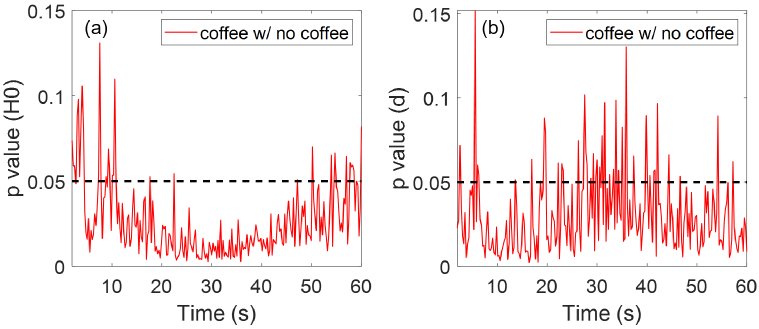
Results of performing the Student’s t-tests on the (a) SC hydration and (b) SC thickness results of the different coffee drinking groups. Here y-axis is the p value and x-axis is the measurement time. The red solid line represents the pairwise comparisons between the two groups as shown in the label. The black dashed line indicates where p=0.05 .

Participants have also been categorised according to whether they have consumed coffee or participated in exercises prior to the experiment. For ease of collecting responses, we have the following options in our questionnaire for coffee and exercise: No, more than 2 hours ago, within the last 2 hours, within the last 1 hour, within the last 30 minutes. Since very few responded with having coffee within the last 30 minutes or 1 hour, we have merged the responses into two groups including participants who had coffee within the last 2 hours and participants who did not have coffee or had coffee more than 2 hours ago. There are 97 participants who have drunk coffee within 2 hours before being measured, and 201 participants who have drunk coffee more than 2 hours before the measurement or haven’t drunk coffee at all. As shown in Fig. 14, the group of participants that have drunk coffee within 2 hours prior to the experiment show higher mean SC hydration and smaller mean SC thickness compared to the other group, with clear separations between their SEM error bars. Therefore, Student’s t-tests were performed on the SC hydration and SC thickness of the two groups, and results in Fig. 15 show that there are significant differences (p<0.05) in the SC hydration and SC thickness between the two groups for most of the measurement time (84% and 83% of the measurement time for SC hydration and SC thickness respectively). This indicates that coffee consumption is likely to be a factor that affects participants’ initial skin hydration and thickness.

**Fig. 16.**
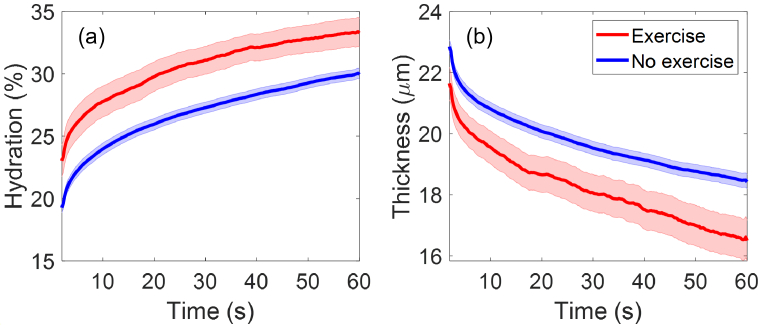
The extracted (a) SC hydration and (b) SC thicknesses as a function of the measurement time compared between participants who have exercised within 2 hours prior to the experiment (31 people) and those who haven’t exercised or have exercised more than 2 hours prior to the experiment (267 people). The solid lines indicate the mean values and the coloured patches show the standard errors of the mean among all the individual results in each category.

**Fig. 17.**
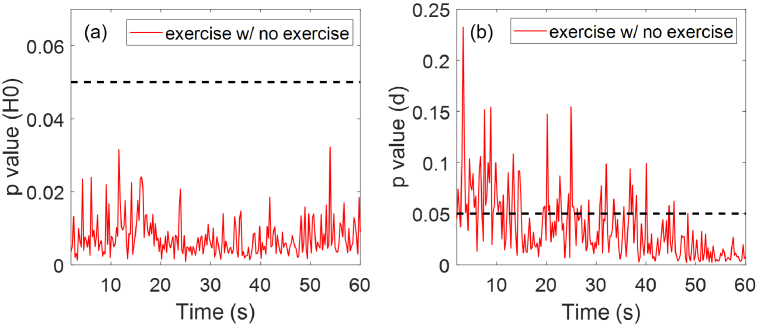
Results of performing the Student’s t-tests on the (a) SC hydration and (b) SC thickness results of the different exercise groups. Here y-axis is the p value and x-axis is the measurement time. The red solid line represents the pairwise comparisons between the two groups as shown in the label. The black dashed line indicates where p=0.05 .

Similarly for participants’ exercise routine, there are 31 participants who have exercised within 2 hours before the measurement, and 267 participants who have exercised more than 2 hours before the measurement or haven’t exercised. Figure 16 shows that the group of participants who have exercised within 2 hours before being measured have higher mean SC hydration and smaller mean SC thickness compared to the other group, with clear separations between their SEM error bars. As shown in Fig. 17, p<0.05 for SC hydration during the entire measurement and p<0.05 for SC thickness for 67% of the measurement time. Therefore, it can be concluded that exercising has a significant impact on participants’ initial skin hydration and also possible impact on the stratum corneum thickness.

## 4. Conclusions

This is the largest THz *in vivo* study of human skin hitherto and the data have been collected across a diverse population. The portable nature of our THz handheld scanner meant that we could set it up in a public space such that we were able to measure over 300 participants in an out-of-the-lab environment. Each participant was measured for 60 seconds, so that we had sufficient data from each participant to observe trends in the dynamic response of skin during the occlusion process and also smooth out any fluctuations due to participants moving during the measurement. The skin hydration profile as a function of time was extracted from the THz spectroscopy data through a model fitting approach with a skin dielectric model. Using the personal information acquired from the questionnaires the participants were categorised into different groups according to their biophysical factors and lifestyle, including sex, age, dominant hand, ethnicity, Fitzpatrick skin type, water intake, coffee consumption and exercise routine. Results show that age, drinking coffee and exercising have significant effects on skin hydration, while influences from other factors such as ethnicity and Fitzpatrick scale are not as clear.

There were no significant differences in hydration or thickness if grouping participants into ethnicity, but if we compared using Fitzpatrick scales there is a slight trend in the SC thickness such that the SC thickness decreases with increasing Fitzpatrick number. The data presented in Fig. 12 are not statistically significant due to the limited sample sizes, but this aspect will be investigated further. It is also noteworthy that many of those with Fitzpatrick type V and type VI responded to the survey to say they had dry skin. Those with dry skin may have applied moisturiser and increased the SC hydration, but this may not have affected the skin thickness, and could explain why the hydration levels when grouped by skin type are closely clustered. In future studies we will investigate if there is a link with melanin content and skin hydration and thickness.

In all of the measurements, the calculated SC thickness decreased with occlusion time. This is in contrast to what we have observed before when we have done measurements using a bench top THz system where for example the SC expanded by nearly 6 *μ*m during 30 minutes of occlusion [32]. However, in that study, the participants placed their arm on the quartz window and each participant effectively controlled the contact pressure, whereas in this study, the hand-held probe is placed onto the participant and the contact pressure is controlled by the operator. We suspect that this caused the skin thickness to be compressed during the measurement. Our future investigations will address this as we will use a robot to hold and control the probe (rather than a person). We are developing such a system, the PicoBot, and we are carefully investigating the effects of contact pressure on the SC thickness. It is also worth noting that the calculated SC hydration increased by about 150% during 60 seconds of occlusion (from about 20% to 30%), whereas the SC thickness typically only decreased by about 20% during the measurement eg from about 23 *μ*m to 19 *μ*m for the “no coffee” group. Thus the most significant changes are in the SC hydration rather than the thickness.

This study has demonstrated the prospective application of *in vivo* THz sensing for real time skin evaluation in a minimally controlled environment, while also providing insight into the types of factors that need to be taken into consideration to achieve a robust protocol. Our future work includes developing the PicoBot to achieve better control of the contact pressure. We will investigate applying THz sensing to evaluate dry skin conditions such as eczema and psoriasis, as well as detection and diagnosis of skin cancer.

## Data Availability

The data presented in this article are publicly available on Figshare [38].
